# Juvenile mucopolysaccharidosis plus disease caused by a missense mutation in *VPS33A*

**DOI:** 10.1002/humu.24479

**Published:** 2022-10-08

**Authors:** Elena V. Pavlova, Dorit Lev, Marina Michelson, Keren Yosovich, Hila Gur Michaeli, Nicholas A. Bright, Paul T. Manna, Veronica Kane Dickson, Karen L. Tylee, Heather J. Church, J. Paul Luzio, Timothy M. Cox

**Affiliations:** ^1^ Department of Medicine University of Cambridge Cambridge UK; ^2^ Wolfson Medical Centre Institute of Medical Genetics Holon Israel; ^3^ The Rina Mor Institute of Medical Genetics Holon Israel; ^4^ The Sackler School of Medicine Tel Aviv University Tel Aviv Israel; ^5^ Department of Clinical Biochemistry, Cambridge Institute for Medical Research, The Keith Peters Building University of Cambridge Cambridge UK; ^6^ Department of Neuroscience and Physiology University of Gothenburg Gothenburg Sweden; ^7^ Willink Biochemical Genetics Unit, Genomic Diagnostics Laboratory, Manchester Centre for Genomic Medicine Central Manchester University Hospitals NHS Foundation Trust St Mary's Hospital Manchester UK

**Keywords:** drug repurposing, endolysosomal acidification, glycosaminoglycan, glycosphingolipid trafficking, intracellular endocytic trafficking, mucopolysaccharidosis, proteasome inhibitor, substrate reduction therapy, VPS33A

## Abstract

A rare and fatal disease resembling mucopolysaccharidosis in infants, is caused by impaired intracellular endocytic trafficking due to deficiency of core components of the intracellular membrane‐tethering protein complexes, HOPS, and CORVET. Whole exome sequencing identified a novel *VPS33A* mutation in a patient suffering from a variant form of mucopolysaccharidosis. Electron and confocal microscopy, immunoblotting, and glycosphingolipid trafficking experiments were undertaken to investigate the effects of the mutant VPS33A in patient‐derived skin fibroblasts. We describe an attenuated juvenile form of VPS33A‐related syndrome—mucopolysaccharidosis plus in a man who is homozygous for a hitherto unknown missense mutation (NM_022916.4: c.599 G>C; NP_075067.2:p. Arg200Pro) in a conserved region of the *VPS33A* gene. Urinary glycosaminoglycan (GAG) analysis revealed increased heparan, dermatan sulphates, and hyaluronic acid. We showed decreased abundance of VPS33A in patient derived fibroblasts and provided evidence that the p.Arg200Pro mutation leads to destablization of the protein and proteasomal degradation. As in the infantile form of mucopolysaccharidosis plus, the endocytic compartment in the fibroblasts also expanded—a phenomenon accompanied by increased endolysosomal acidification and impaired intracellular glycosphingolipid trafficking. Experimental treatment of the patient's cultured fibroblasts with the proteasome inhibitor, bortezomib, or exposure to an inhibitor of glucosylceramide synthesis, eliglustat, improved glycosphingolipid trafficking. To our knowledge this is the first report of an attenuated juvenile form of VPS33A insufficiency characterized by appreciable residual endosomal‐lysosomal trafficking and a milder mucopolysaccharidosis plus than the disease in infants. Our findings expand the proof of concept of redeploying clinically approved drugs for therapeutic exploitation in patients with juvenile as well as infantile forms of mucopolysaccharidosis plus disease.

## 1 INTRODUCTION

A fatal lysosomal disease resembling mucopolysaccharidosis, caused by a missense mutation in *VPS33A* gene NM_022916.5:c.1492C>T (c.1492C>T); NP_075067.2:p.Arg498Trp (p.Arg498Trp), has recently been identified in infants of Turkish and Yakutian ethnic background: this has been named mucopolysaccharidosis plus (MPSPS; MIM# 610034) (Dursun et al., 2017; Kondo et al., 2017; Pavlova et al., 2019). The mutation was found in homozygous form in two infant siblings born to a consanguineous Turkish family (Dursun et al., 2017) and in 17 young children in the Republic of Sakha (Yakutia), an ethnically Turkic population originating from nomadic people who migrated across the Balkans and Central Asia (Kondo et al., 2017; Pavlova et al., 2019; Vasilev et al., 2020). The disease is apparent in early infancy with manifestations suggesting a mucopolysaccharidosis: coarse features, multiple dysostosis, breathing difficulties, joint contractures, impaired motor development, diffuse muscle hypotonia, ataxia, and inability to stand or walk. Of note, however, systemic manifestations shared with the Chédiak−Higashi, Hermansky−Pudlak, and Griscelli syndromes that affect lysosome‐related organelles occur: these include broncho‐pulmonary complications, severe renal impairment, susceptibility to infections, defective immunity associated with neutropenia, and appearance of azurophilic granules in peripheral lymphocytes, retinal hypopigmentation, nystagmus, and a bleeding tendency. Hitherto, all Yakutian patients reported with this disease in infancy have died by the age of 4 years (Kondo et al., 2017; Pavlova et al., 2019; Vasilev et al., 2020), the Turkish patients died before they were 6 years old (Dursun et al., 2017). In consideration of the characteristic clinical features of mucopolysaccharidosis, biochemical investigations revealed pathological excess of urinary glycosaminoglycans (GAG) and sialylated conjugates while activities of the cognate lysosomal hydrolases responsible for degradation of these complex mucopolysaccharides were unimpaired. In culture, fibroblasts from the patients showed increased vacuolation and expansion of the lysosomal compartment. Defective endocytic trafficking of glycosphingolipids was also identified (Pavlova et al., 2019),—a feature typical of sphingolipidoses regardless of their genetic cause (Marks et al., 2013; Pagano, 2003; Pryor et al., 2006; Puri et al., 1999; Vruchte et al., 2004).

The disease is caused by a reduced abundance of vacuolar protein sorting 33A protein (VPS33A), a component of two intracellular, hexameric, membrane‐tethering, protein complexes, the homotypic fusion and vacuole protein sorting complex (HOPS), and the class C core vacuole/endosome tethering complex (CORVET). These complexes regulate SNARE assembly and tether endosomes and lysosomes in the endocytic pathway (Balderhaar & Ungermann, 2013; Bröcker et al., 2012; Perini et al., 2014; Seals et al., 2000). Genetic deficiency of the HOPS/CORVET subunit VPS16 has been found to phenocopy mucopolysaccharidosis plus disease (Sofou et al., 2021).

We showed that treatment with a proteasome inhibitor rescued the mutant VPS33A^p.Arg498Trp^ from proteasomal degradation in experimental cell lines expressing the protein variant (Pavlova et al., 2019). We explored the effects of two clinically approved drugs on the abnormal late endocytic trafficking in patient‐derived fibroblasts: (i) the proteasome inhibitor, bortezomib, to counter the premature destruction of unstable mutant VPS33A molecules and (ii) an inhibitor of glucosylceramide biosynthesis with the UDP‐glucose: N‐acylsphingosine d‐glucosyltransferase (UGCG), eliglustat tartrate (Pavlova et al., 2019). Each treatment substantially improved defective intracellular trafficking of glycosphingolipids and thus offer a potential therapeutic opportunity in this lethal orphan disease (Pavlova et al., 2019). Moreover, should substrate reduction with eliglustat (Cox, 2010; McEachern et al., 2007; Shayman, 2015) ameliorate the systemic disease, an opportune case for using brain‐penetrant analogues (Fujii et al., 2021; Marshall et al., 2016) to address the neurodegenerative manifestations of the MPS plus would be greatly strengthened.

For the first time here we describe a unique VPS33A‐related cellular trafficking syndrome‐mucopolysaccharidosis plus in an adult who is homozygous for a hitherto unknown missense mutation (NM_022916.4: c.599 G>C; p.Arg200Pro) in a conserved region of the *VPS33A* gene. Based on the age of presentation, longevity, absence of severe systemic complications and susceptibility to infections, biochemical, and cellular pathology in the context of the variant, we contend that the mutation is associated with an attenuated juvenile form of the mucopolysaccharidosis plus disease.

## 2 MATERIALS AND METHODS

### 2.1 Patients

The patient, a young adult, was referred from the Institute of Medical Genetics, Wolfson Medical Center, Holon, Israel in July 2019, where the genetic diagnosis was first made (D. L., M. M., K. Y., M. G.). We previously reported the clinical characteristics of five Yakutian patients (Pavlova et al., 2019). Retrospective non‐identifiable clinical data were analyzed according to local and international ethical regulations. Informed consent was obtained from the parents of the patients in accordance with the standards of the Declaration of Helsinki. The study in the present case was approved by the Ethics Committee of The Institute of Medical Genetics, Wolfson Medical Center, Israel. Parents of the patient consented for publication.

Detailed information about the samples and biochemical analyses is described in Supporting Information.

### 2.2 Next generation exome and Sanger sequencing

The description of sequencing analysis is provided in Supporting Information.

### 2.3 Cell culture and immunoblotting

Healthy adult and neonatal fibroblasts were obtained from Sigma (Catalogue numbers 106‐05A; 106‐05N) and normal adult fibroblasts from Lonza (Catalogue number CC2511). Patients' and healthy control dermal fibroblasts were cultured in Minimum Essential Medium Eagle (Sigma) or nutrient media F10 supplemented with 10%−20% fetal bovine serum (Sigma), 1 mM sodium pyruvate (Sigma), 2 mM Glutamax‐1 (Gibco), 100 IU/ml penicillin, 100 µg/ml streptomycin (Invitrogene) at 37°C in a humidified atmosphere of 5% CO_2_.

Patients' and control fibroblast cell lines were tested and confirmed to be free of mycoplasma species by Eurofins Mycoplasma service.

Methods used for immunoblotting are supplied in Supporting Information.

### 2.4 Electron microscopy

The detailed descriptions for horseradish peroxidase (HRP) uptake to flood the fibroblast endocytic system, fixation, staining, and cutting of ultrathin sections are provided in the Supporting Information. For stereological analysis, 50 images per sample were collected at a magnification of x890 using an unbiased raster pattern (Griffiths, 1993). The volume fraction in individual cell profiles of the cell cytoplasm was determined by point counting using a 10 μm^2^ grid overlay and the volume fraction of endocytic organelles containing HRP was determined using a 2 μm^2^ grid overlay using NIH ImageJ (Version 2.0.0‐rc‐69/1.52i).

### 2.5 Confocal microscopy

Cells were grown on 4 well glass slides (Millipore) in normal growth medium for 24 h. After fixation the cells were, permeabilized using the fixation/permeabilization kit (BD Biosciences catalogue 554714) and incubated with primary mouse anti‐human LAMP2 (Abcam) or EEA‐1 (BD Biosciences) monoclonal antibodies and an Alexa Fluor 488 conjugated goat anti‐mouse IgG (H + L) antibody. The slides were mounted using ProLong Gold antifade Mountant with DAPI nuclear stain (Thermo Fisher Scientific). The slides were examined using a Leica Sp5 confocal microscope and serial 0.3−0.5 µm Z‐stacks images per field (5−7 cells) were acquired. To analyse acidic lysosomes in situ, the cells were incubated with LysoTracker red DND‐99 (Thermo Fisher Scientific) and examined by confocal imaging. Images were analyzed using ImageJ software.

### 2.6 Lactosyl ceramide trafficking

Cells were incubated with 5 µM BSA‐BODIPY‐C_5_lactosylceramide(LacCer) as previously described (Pavlova et al., 2019) (Supporting Information).

## 3 RESULTS

### 3.1 Clinical and biochemical characteristics of an adult patient with mucopolysaccharidosis plus disease

**Figure 1:**
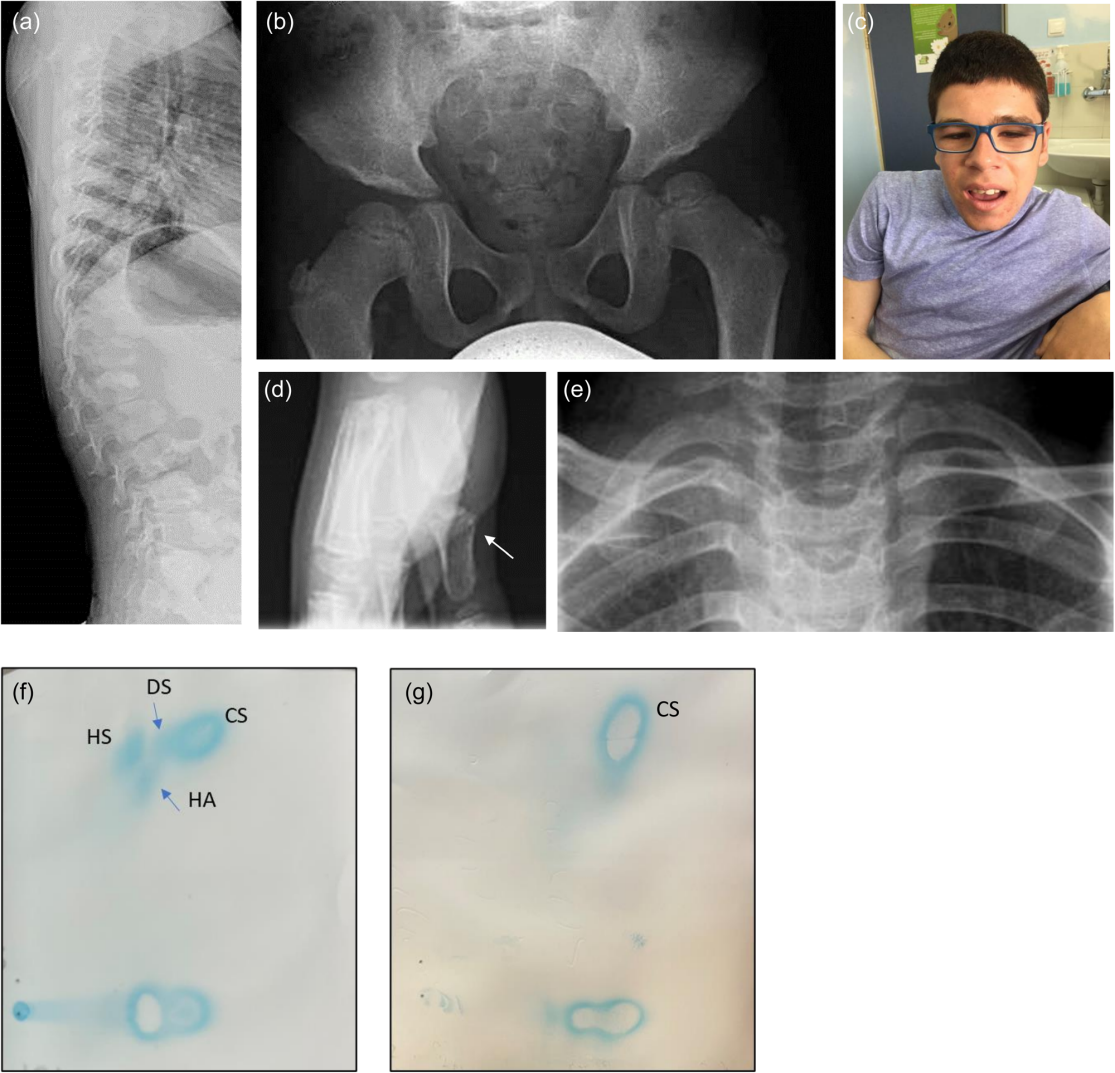
Skeletal complications and two‐dimensional electrophoresis of urinary GAGs in patients with a juvenile form of mucopolysaccharidosis plus disease. (a) Lateral spinal radiograph of patient P6 in middle childhood showing flattened and oval‐shaped vertebrae. (b) Radiograph of pelvic showing flattening acetabula obtained in early childhood. (c) Patient P6 in late adolescence showing coarse facial features, ptosis, short neck. (d) Radiograph of foot in early childhood: widening of metatarsal bones. (e) Broad and short claviculae on cervical spine radiograph of patient P6 in late childhood. (f) Patient P6 urinary GAG pattern showed increased heparan (HS), some dermatan (DS) sulphates, and hyaluronic acid (HA). (g) Healthy individual with normal pattern of GAG. GAG, glycosaminoglycan.

The patient [designated Patient P6], a young adult of Southern European/Mediterranean origin has been clinically monitored since the age of 2.5 years when psychomotor developmental delay was noted. Abdominal examination revealed hepatosplenomegaly and proteinuria in early childhood. The disease progressed slowly: ptosis, coarse features, skeletal deformities including retrognathia and marked kyphoscoliosis developed by the age of 6 years, (Figure 1a−e; Table 1). The patient had a moderate intellectual disability characterized by difficulties in concentration and attention, poor memory, and behaviors that reflect autism spectrum syndrome. Radiological examination revealed multiple skeletal deformities including flattening of vertebrae, acetabula, and widening of metatarsal bones (Figure 1a−e). Transthoracic echocardiogram revealed thickening of the interventricular septum and a hyperechogenic aortic signal. Semiquantitative analysis of urinary GAG by two‐dimensional electrophoresis on a specimen obtained in adulthood, showed increased heparan and dermatan sulphate as these had previously been recorded in patients, designated patients P1 and P2, harboring the p.Arg498Trp mutation in the VPS33A protein (Pavlova et al., 2019) (Figure 1f). In addition, patient P6 had increased urinary hyaluronic acid—a non‐sulphated GAG which was not clearly observed in samples of three Yakutian patients with the p.Arg498Trp mutation (Pavlova et al., 2019). Urine sialylated conjugates and oligosaccharides were not increased. Enzymatic activities of relevant lysosomal hydrolases were normal, thereby, excluding diagnosis of MPS I and II, III A, B, C, D, MPSVI, GM1 and GM2 gangliosidoses, Pompe disease, and sialidosis.

**Table 1:** Clinical signs and features in patients with mucopolysaccharidosis plus disease

	Infantile form		Juvenile form
Signs/symptoms Gene, mutation	VPS33A	VPS16	VPS33A
	p.Arg498Trp^a^ (Dursun et al., 2017; Pavlova et al., 2019)	c.2272‐18C>A^b^ (Sofou et al., 2021)	p.Arg200Pro^c^
Number of patients	*n* = 7	*n* = 2	*n* = 1
Age/onset	Infancy	Infancy	Early‐middle childhood
Coarse features	6/7	2	+
Recurrent respiratory, intestine infections	7/7	2	−
Susceptibility to infections	7/7	2	−
Splenomegaly	6/7	2	In childhood
Neutropenia	4/5	2	−
Anemia	7/7	2	−
Sepsis	1/5		−
Thrombocytopenia	7/7	2	−
Coagulation defect	2/5	‐	−
Recurrent bronchopneumonia and chronic obstructive pulmonary disease	7/7	2	−
Motor developmental delay	7/7	2	+
Delayed speech development	7/7	2	+
Dysostosis multiplex	7/7	2	+
Joints contraction	7/7	2	+
Nephrotic syndrome	4/7	‐	−
Hypoalbuminemia	5/7	‐	−
Hyperproteinuria	7/7	‐	In childhood
Hypopigmentation of retina	1/7	‐	−
High serum IgM concentration	4/7	‐	−
Low serum IgG concentration	4/7	‐	−
Hypotonia	7/7	2	−
Poor tendon reflexes	3/5	‐	−
Myelination delay	1/5	2	
Nystagmus	1/5	‐	−
Intellectual disability		‐	+
Autism spectrum syndrome	‐	‐	+
Kyphoscoliosis	1/5	‐	+
Accumulation of dermatan and heparan sulphate	7/7	2	+
Increased sialic conjugates	2/5	2	−
Hyaluronic acid	‐	‐	+
Age/age at death	Infancy—early childhood	Early childhood	Alive early 20s
Ethnicity	Turkish, Yakutian	Turkish, Iranian	Turkish‐Jewish‐Bulgarian

^a^ NM_022916.5: c.1492 C>T(p.Arg498Trp).

^b^ NM_022575.3: c2272‐18 C>A.

^c^ NM_022916.4:c.599G>C (p.Arg200Pro).

### 3.2 Next generation and Sanger sequencing analysis

Next generation exome sequencing of the DNA from patient P6 identified a missense variant NM_022916.4: c.599 G>C; NP_075067.2: p.Arg200Pro in the *VPS33A* gene. This caused replacement of arginine by proline at position 200 of the VPS33A protein (p.Arg200Pro). Sanger sequencing analysis confirmed that the patient was homozygous and both parents were heterozygous carriers (Supporting Information: Figure S1). In addition, the patient's healthy brother and sister were heterozygous carriers, indicating autosomal recessive inheritance.

### 3.3 Endosomal/lysosomal impairment caused by mutations p.Arg200Pro and p.Arg498Trp in VPS33A

**Figure 2:**
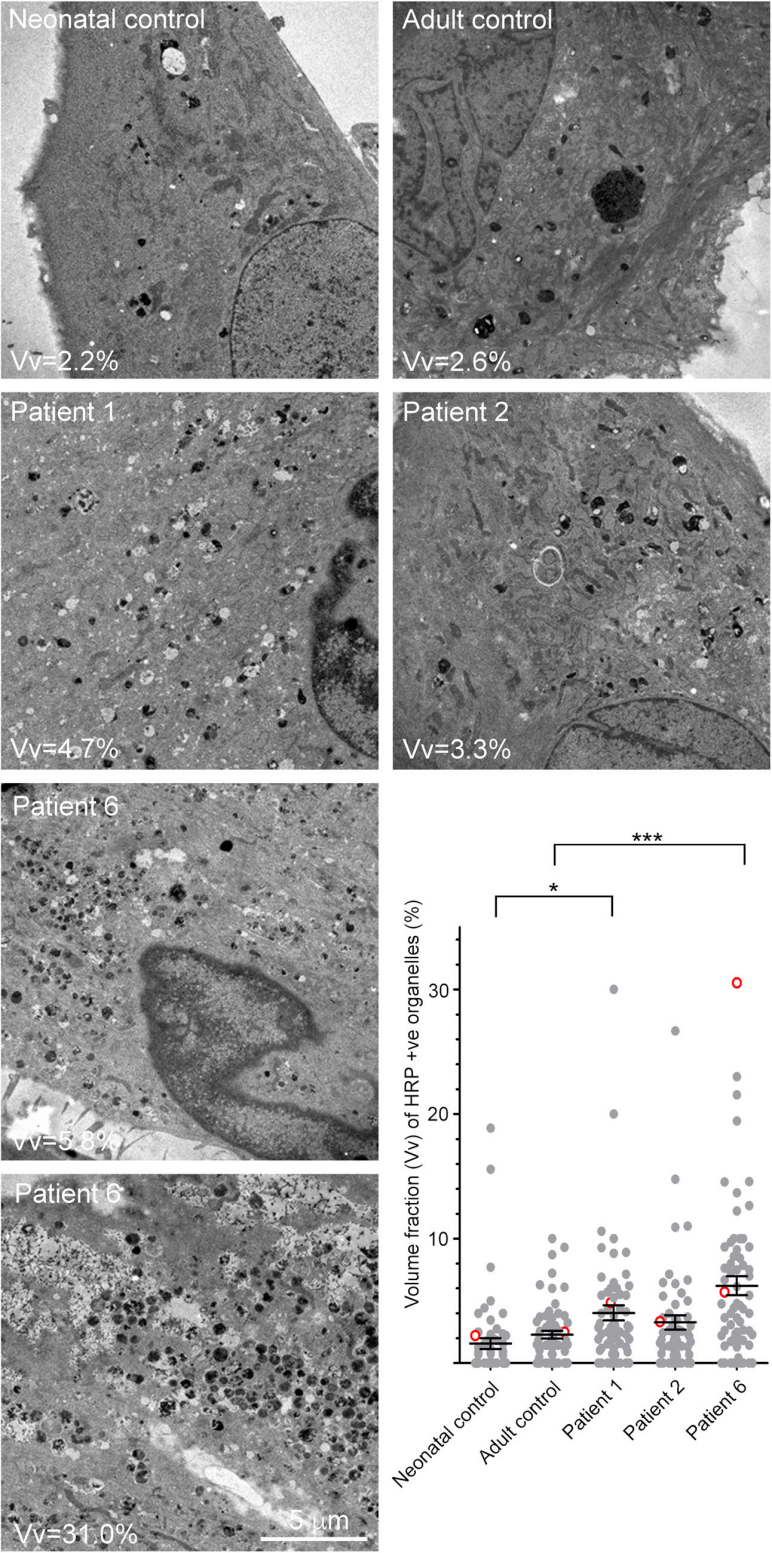
HRP positive endocytic organelles in patient‐derived fibroblasts. Electron micrographs of thin sections of control and patient‐derived fibroblast cells which had endocytosed medium containing 2 mg/ml HRP continuously for 24 h to flood the endocytic system. The volume fraction (Vv) occupied by HRP +ve organelles is shown on each micrograph. The graph shows the volume fraction of HRP +ve organelles in >60 cell profiles in control and patient‐derived fibroblasts and the open circles (red border) indicate the position within the data set of the representative electron micrographs shown in this figure. HRP, horseradish peroxidase.

To investigate intracellular morphological changes, skin fibroblasts from the VPS33A^R200P^ patient P6 were cultured in vitro. We observed enlarged vacuoles by electron microscopy of the VPS33A^p.Arg200Pro^ patient‐derived fibroblasts as we had previously reported in fibroblasts from VPS33A^p.Arg498Trp^ patients P1 and P2 (Pavlova et al., 2019). To quantify the enlargement of compartments along the whole endocytic pathway (i.e., all endosomes, endolysosomes, and lysosomes), we allowed the cells to endocytose HRP continuously for 24 h; the volume fraction of HRP‐laden endocytic organelles in electron microscopy images of individual cells was determined by a lattice overlay method of stereological sampling (see Section 2; Figure 2 and Supporting Information: Figure S2). We compared the volume fraction of HRP‐positive endocytic organelles in the cytoplasm of the VPS33A^p.Arg200Pro^ fibroblasts (P6, mean 6.2 ± 0.8%) with the volume fraction in fibroblasts from two VPS33A^p.Arg498Trp^ patients (P1, 4.0 ± 0.6%; P2, 3.3 ± 0.6%) as well as neonatal (1.6 ± 0.4%) and adult (2.3 ± 0.3%) control fibroblasts (Figure 2). These data confirmed the enlargement of endocytic organelles in fibroblasts of VPS33A^p.Arg498Trp^ patients, previously inferred from counts of enlarged vacuoles: comparable enlargement was found in the cells from the patient with the VPS33A^p.Arg200Pro^ variant.

**Figure 3:**
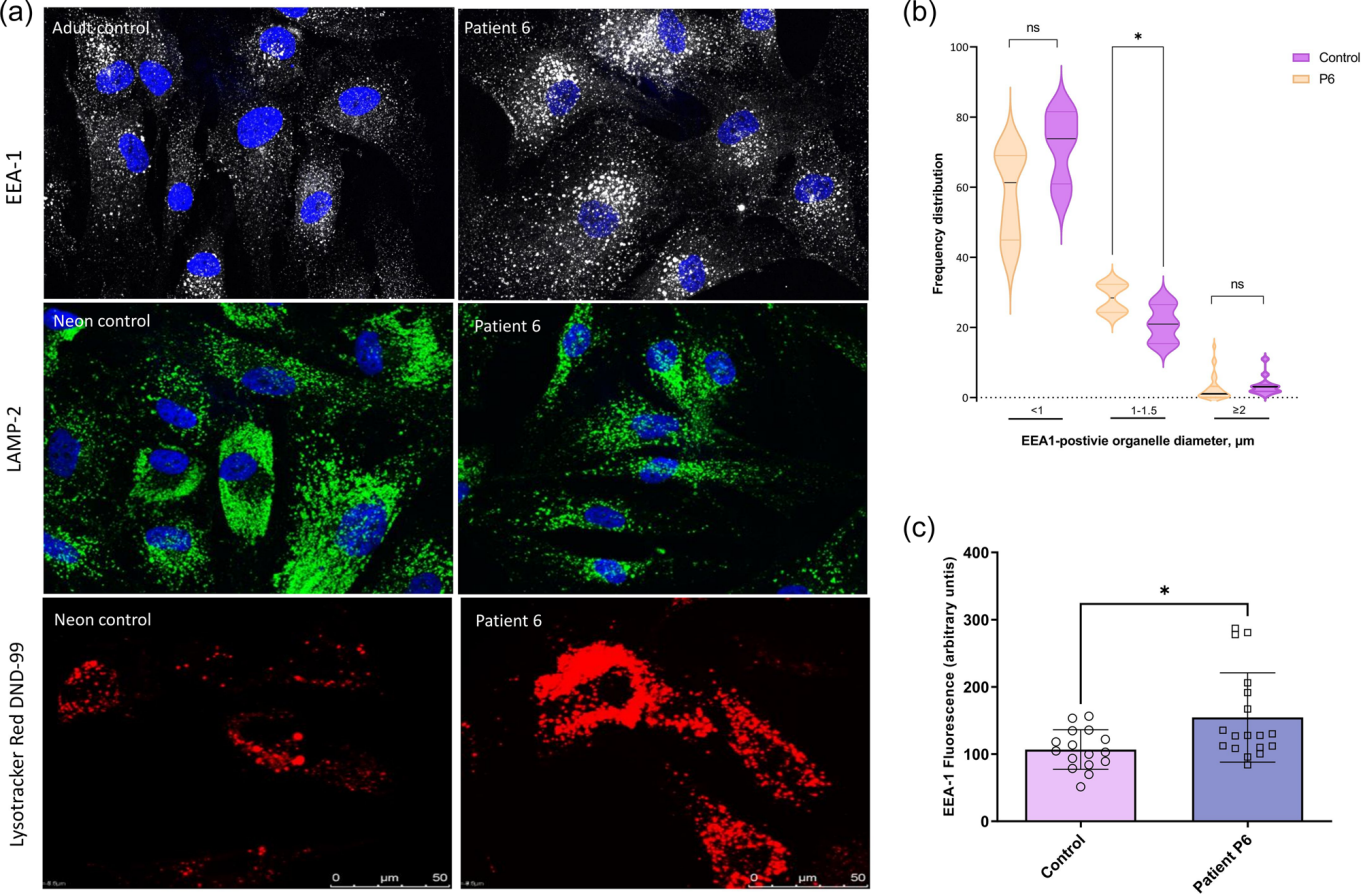
Early endosomal antigen (EEA1) and late lysosomal markers in patient‐derived fibroblasts. (a) Representative confocal images of cultured patient P6 and control fibroblasts stained with early endosomal marker EEA1, lysosome‐associated membrane protein LAMP2 antibody and secondary anti‐human IgG Alexa‐488 (white or green respectively) and DAPI (blue); representative confocal images of patient P6 and neonatal control fibroblasts show increased acidotropic probe LysoTracker red DND‐99 fluorescence intensity. (b) Distribution of EEA1 positive organelles accordingly the puncta diameter bin <1, 1−1.5, and >2 µm quantified in six cells of each control and P6 fibroblasts. Violin graphs show the median and quartiles of the quantified EEA1 positive organelles. (c) Integrated density of EEA1 fluorescence in the patient P6 and control cells. *p< 0.05. The fluorescence intensity per cell was quantified in images of maximum intensity Z‐projections. The cellular area, the integrated density, and the mean gray values were analyzed. Measurements of regions without fluorescence were used for background subtraction. The net average fluorescence intensity per pixel, expressed as corrected total cell fluorescence, was calculated for each cell.

Further evidence of altered structure and function of the endocytic compartments in VPS33A^p.Arg200Pro^ cells was obtained by fluorescence microscopy. Total immunofluorescence of early endosomal antigen 1 (EEA1) was markedly increased in VPS33A^p.Arg200Pro^ patient‐derived fibroblasts (P6, Figure 3a,b) as had previously been observed in VPS33A^p.Arg498Trp^ fibroblasts derived from patients P1 and P2 (Pavlova et al., 2019). In addition, quantification of EEA1 positive puncta showed a significantly increased proportion of EEA1 positive organelles with an enlarged diameter (1−1.5 µm) in the VPS33A^p.Arg200Pro^ patient's fibroblasts compared with control cells (Figure 3c). Conversely, the proportion of EEA1 positive puncta with a diameter less than 1 µm was greater in control fibroblasts (Figure 3c). In contrast to EEA1, but in agreement with previous data from VPS33A^p.Arg498Trp^ patient‐derived fibroblasts (Pavlova et al., 2019), the fluorescence intensity of the lysosomal marker, LAMP2, was not significantly different from that in control fibroblasts (Figure 3a). Incubation of the VPS33A^p.Arg200Pro^ patient‐derived fibroblasts with the acidotropic dye LysoTracker Red DND‐99, resulted in increased total fluorescent intensity in VPS33A^p.Arg200Pro^ fibroblasts (Figure 3a) compared with control cells, as previously reported for LysoSensor blue dextran DND‐167 in VPS33A^p.Arg498Trp^ fibroblasts (Kondo et al., 2017; Pavlova et al., 2019). This is consistent with a lower pH of the endosomal/lysosomal compartments in the patient‐derived cells. Taken together, the findings on microscopy indicate altered morphology and dysfunction of endosomal/lysosomal compartments in fibroblasts derived from patients with either VPS33A mutation.

### 3.4 The effect of substitution of arginine 200 by proline on VPS33A protein stability

**Figure 4:**
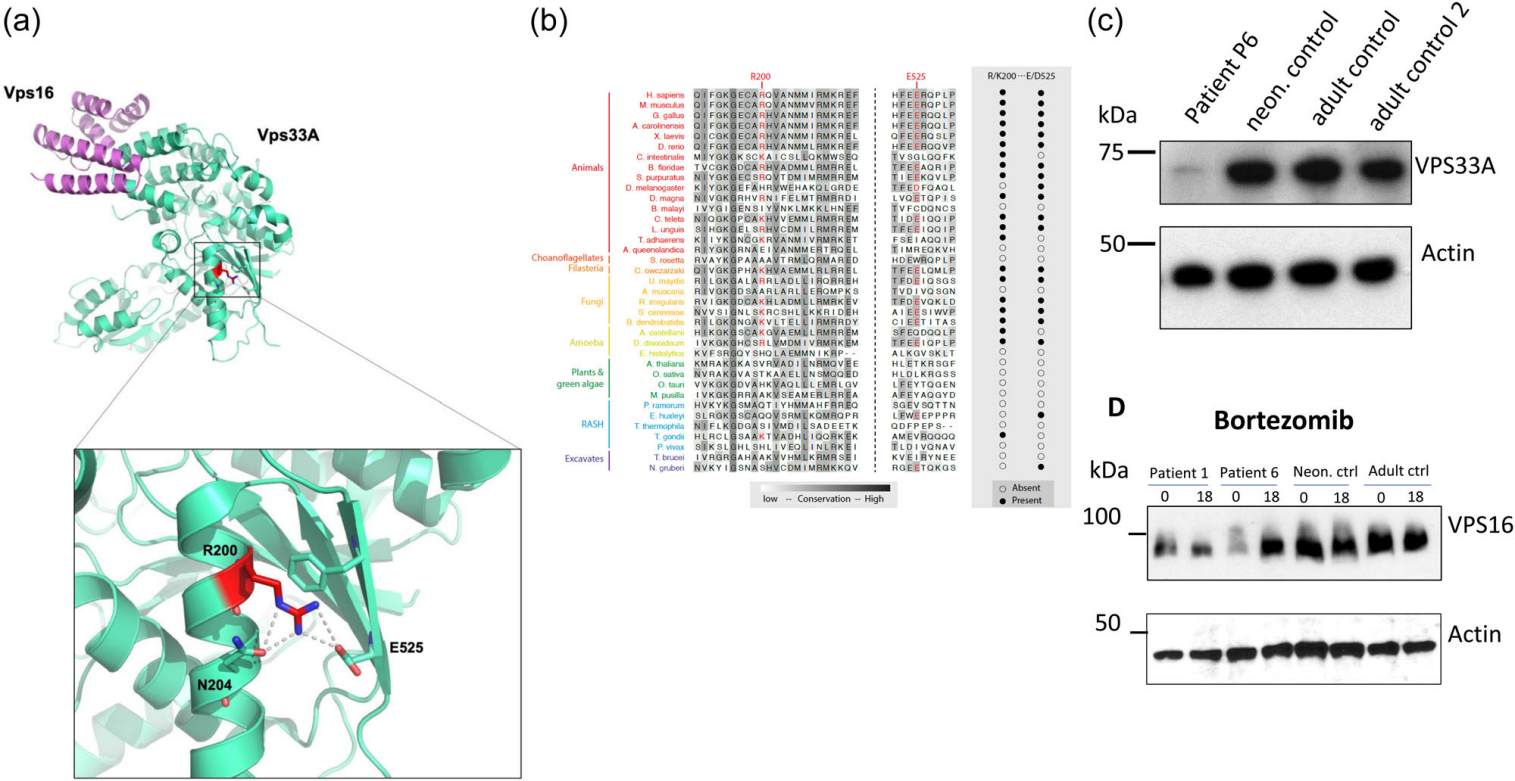
The p.Arg200Pro mutation affects stability of the VPS33A protein. (a) Cartoon representation of the crystal structure of human VPS33A (green) in complex with VPS16 (Graham et al., 2013; Wartosch et al., 2015). Arginine 200 (highlighted red) is located in a helix and forms a salt bridge with glutamic acid 525. (b) Phylogenetic tree and alignment of VPS33A sequences shows conservation of arginine 200 and glutamic acid 525 residues in vertebrates and amongst species from amoebae to higher vertebrates. (c) Immunoblot analysis showing reduced abundance of VPS33A protein in patient P6 fibroblasts compared to neonatal (neon) and two adult control fibroblasts. (d) In fibroblasts of patient P1 with p.Arg498Trp and patient P6 with p.Arg200Pro mutated VPS33A, reduced protein abundance was also observed for the HOPS/CORVET subunit VPS16. Actin was used as a loading control. Fibroblasts of patient P1 and patient P6 were incubated with 100µM bortezomib (PS‐341; Stratech; catalogue number S1013) for 18h in normal cell culture media (MEM supplemented with 10% [v/v] FCS), (2 mM glutamine, 100 U/ml penicillin, and 100 μg/ml streptomycin) and cell lysates analyzed by immunoblotting with polyclonal rabbit anti‐human VPS16 antibody and secondary HRP‐conjugated anti‐rabbit IgG or anti‐actin antibodies. HRP, horseradish peroxidase; MEM, Minimum Essential Medium Eagle.

Examination of the crystal structure of human VPS33A in complex with a fragment of VPS16 (Graham et al., 2013) showed that the residue arginine 200 is localized in the same domain 2 as the residue arginine 498 (Supporting Information: Figure S3). Arginine 200 is in a helix and forms an intramolecular salt bridge with glutamic acid 525 (Figure 4a). Replacement of arginine 200 by proline is predicted to disrupt the helix, salt bridge, and local ionic network, resulting in destablization of the folded protein thereby accelerating proteasomal degradation. Interspecies alignment analysis of the protein sequence showed that the arginine 200 and glutamic acid 525 residues are preserved in vertebrates with evidence of evolutionary conservation from amoebae to vertebrates (Figure 4b).

Immunoblotting of protein lysates obtained from patient‐derived VPS33A^p.Arg200Pro^ fibroblasts revealed a decreased abundance of full‐length (65 kDa) VPS33A compared with three healthy control fibroblast lysates (Figure 4c). These data are consistent with substitution of residue arginine 200 by proline resulting in reduced protein stability and proteolysis. Destabilization of VPS33A is predicted to impair assembly/stability of the CORVET and HOPS complexes (Wartosch et al., 2015). In support of this argument, abundance of the CORVET/HOPS component VPS16 as detected by immunoblotting was also reduced in lysates obtained from VPS33A^p.Arg498Trp^ and VPS33A^p.Arg200Pro^ (P1 and P6, respectively) lysates (Figure 4d). Moreover, incubation of patient‐derived fibroblasts with a proteasome inhibitor, bortezomib, for 18 h, increased the steady state concentration of VPS16 in the lysates close to that in control cells (Figure 4d).

### 3.5 Delayed BODIPY‐Lactosylceramide trafficking in VPS33A^p.Arg200Pro^ patient derived fibroblasts

**Figure 5:**
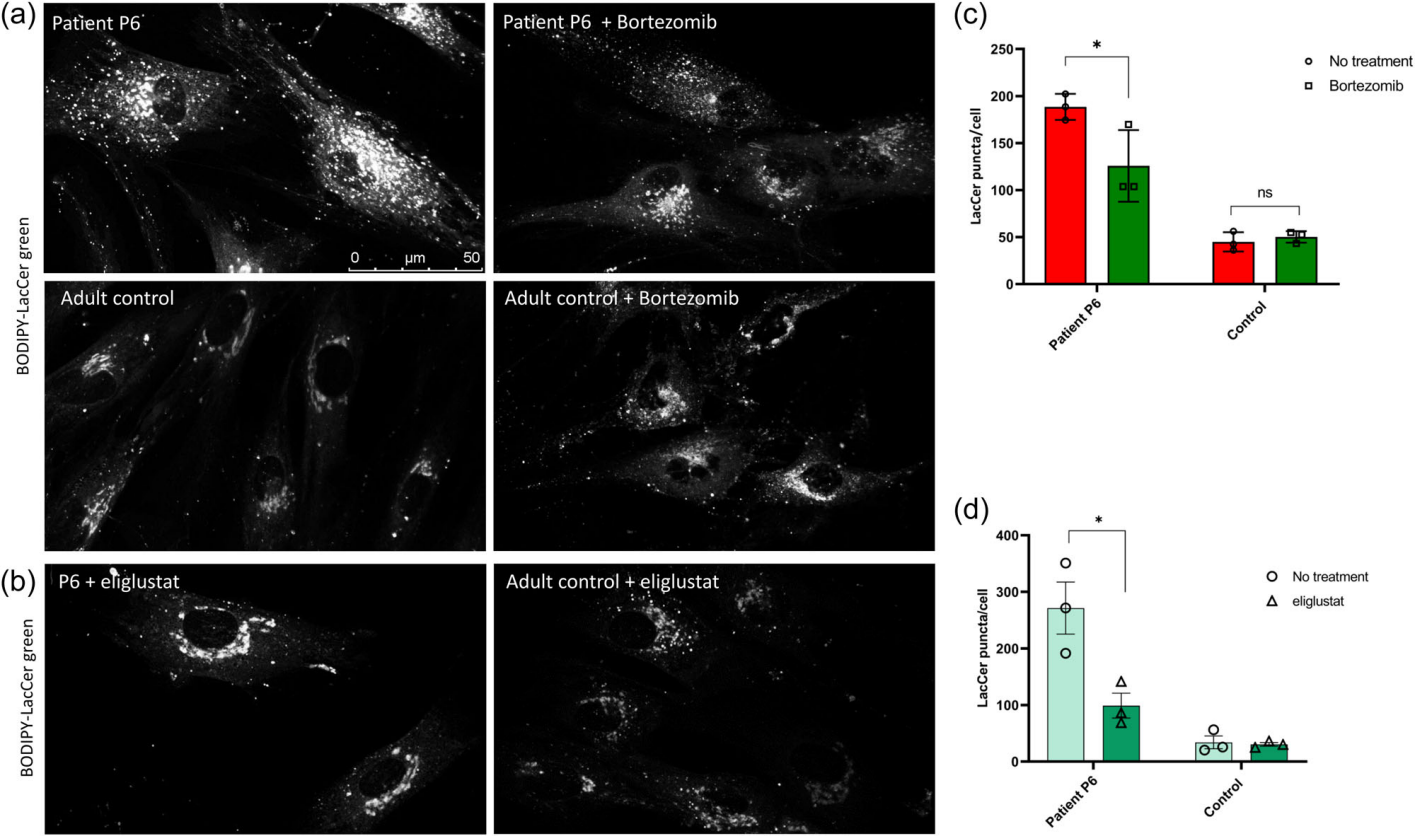
Abnormal BODIPY‐LacCer trafficking in patient‐derived fibroblasts is corrected by proteasome inhibitor bortezomib and improved by inhibitor of β‐glucosylceramide synthesis, eliglustat tartrate. Patient‐derived P6 or control fibroblasts were grown on 35 mm glass‐bottom tissue culture dishes in normal media supplemented with 100 µM of bortezomib (PS‐341; Stratech; catalogue number S1013) for 18 h. (a) Fibroblasts were labeled with BODIPY‐LacCer and endocytosed BODIPY‐LacCer was observed after 3 h uptake. (b) Patient‐derived P6 and control fibroblasts were incubated with a specific inhibitor of UDP‐glucosylceramide synthase, 50 nM eliglustat tartrate for 24 h. Representative images of patient‐derived cells and control cells labeled with BODIPY‐LacCer and observed on confocal microscope. (c) Quantification of BODIPY‐LacCer puncta in the patient‐derived P6 and control fibroblasts was performed in ≥10 cells per each line from three independent bortezomib experiments. Data presented as mean ± SEM, *p< 0.05, NS, not significant. Patient‐derived and control fibroblasts were incubated with a specific inhibitor of UDP‐glucosylceramide synthase, 50 nM eliglustat tartrate for 24 h. (d) Quantification of BODIPY‐LacCer puncta in patient P6 and control fibroblasts in ≥10 cells from three independent eliglustat experiments. 5−10 single live cell images (each containing 3−5 cells) were analyzed using ImageJ software. For each image, an 8‐bit gray scale image was generated using thresholds removing background signals. Pixels clearly corresponding to Golgi fluorescence were selected and removed from the images and puncta containing the remaining pixels were counted. Data presented as mean ± SEM, *p< 0.05.

We previously reported a delayed trafficking of endocytosed BODIPY‐Lactosylceramide C_5_‐lactosylceramide (BODIPY‐LacCer) in fibroblasts obtained from patients with VPS33A^p.Arg498Trp^ (Pavlova et al., 2019). To investigate whether VPS33A^p.Arg200Pro^ has a similar effect on lactosylceramide trafficking, we conducted BODIPY C_5_‐lactosylceramide (BODIPY‐LacCer) pulse‐labeling and chase experiments in fibroblasts from patients and control fibroblasts (Pavlova et al., 2019; te Vruchte et al., 2004). As shown in VPS33A^p.Arg498Trp^ fibroblasts (Pavlova et al., 2019), when compared with the signal in control fibroblasts BODIPY fluorescence in the VPS33A^p.Arg200Pro^ patient P6 cells was observed in more peripheral punctate structures, consistent with its accumulation in endocytic organelles (Figure 5). As previously reported in fibroblasts of VPS33A^p.Arg498Trp^ patients, quantification of BODIPY‐LacCer fluorescence confirmed that there were fewer peripheral puncta in the VPS33A^p.Arg200Pro^ patient's cells exposed to treatment with bortezomib (100 µM, 18 h) (Figure 5a). This delayed glycosphingolipid trafficking in patient P6 fibroblasts was partially corrected by treatment with the protease inhibitor in three independent experiments (Figure 5c).

### 3.6 Substrate reduction therapy improves lactosylceramide trafficking in VPS33A^p.Arg200Pro^ patient‐derived cells

To investigate if reduction of glycosphingolipid biosynthesis could improve the endosomal‐lysosomal recycling of fluorescent lactosylceramide, we treated patient P6 fibroblasts with 50 nM eliglustat and miglustat, N‐butyldeoxynojirimycin, at micromolar concentrations for 24 h as previously described (Pavlova et al., 2019) and pulse‐chase labeled the fibroblasts with BODIPY‐LacCer. Also, as was previously observed with VPS33A^p.Arg498Pro^ patients' fibroblasts, exposure to 50 nM eliglustat and 100 µM miglustat, redistributed BODIPY fluorescence principally to the Golgi area (Figure 5b and data not shown). This indicates that inhibition of de novo glycosphingolipid biosynthesis reduces intracellular accumulation of glucosylceramide and improves endocytic trafficking in VPS33A^p.Arg200Pro^ fibroblasts (Figure 5b,d).

## 4 DISCUSSION

The recently recognized class of membrane trafficking disorders (García‐Cazorla et al., 2022) such as that due to VPS33A mutations—mucopolysaccharidosis plus, [caused by deficiency of the HOPS complex], has pleiotropic clinical features. The infantile form of VPS33A‐related mucopolysaccharidosis plus disease manifests in the first year of life with delayed psychomotor development, failure to thrive, diffuse muscle hypotonia, joints contractures, dysostosis multiplex, and rapid progression of multisystem disease affecting respiratory, immune, cardiovascular, hematological, gastrointestinal, renal, and central nervous systems. Patients suffer life‐threatening episodes of sepsis related to immunodeficiency and often require support that includes assisted ventilation, parenteral nutrition, and blood transfusions. Patients with the infantile VPS33A‐related disease died with multisystem failure triggered by infection within the first six years of life. Here we describe an attenuated juvenile onset form of mucopolysaccharidosis plus that is due to inheritance of a previously unreported homozygous variant p.Arg200Pro in VPS33A protein, a core subunit of the HOPS and CORVET tethering complexes identified in an adolescent patient with long‐standing intellectual disability, skeletal deformities, and evidence of mild cardiac disease (Dursun et al., 2017; Kondo et al., 2017; Pavlova et al., 2019).

Of note, two patients have been lately reported with an overlapping phenocopy of mucopolysaccharidosis plus disease caused by genetic deficiency of VPS16, another subunit of the HOPS and CORVET tethering complexes (Sofou et al., 2021) to which VPS33A is recruited by binding. These patients had progressive psychomotor regression, delayed myelination, and mucopolysaccharidosis‐like features apparent in infancy (Table 1). Both patients were homozygous for the same intronic variant (NM_022575.3: c.2272‐18C>A) in *VPS16*, that generates a hypomorphic allele causing the deficiency of both subunits VPS16 and VPS33A. These patients also had marked systemic manifestations in common with infants with the p.Arg498Trp mutation in VPS33A and features shared with Chédiak−Higashi syndrome including neutropenia, splenomegaly, thrombocytopenia, vacuolised peripheral lymphocytes, and dense azurophilic granules in myeloid progenitor cells of bone marrow with susceptibility to infections. Patient‐derived fibroblasts showed defective endosomal trafficking of transferrin and accumulation of autophagosomes (Sofou et al., 2021). In this connection, the development of a steroid‐responsive acute haemophagocytic histiolymphocytosis recently reported in an infant of Moroccan ethnic background with mucopolysaccharidosis plus and the identified p.Arg498Trp mutation in VPS33A, merits attention. The response to intensive corticosteroid therapy is typical of an impaired innate immunity and possible autoinflammation triggered by infection in patients with mucopolysaccharidosis plus disease (Faraguna et al., 2022).

Inborn errors of abnormal endocytic trafficking caused by deficiency of VPS33A, VPS16, and potentially all other subunits of the HOPS complex are likely to affect biogenesis of lysosome‐related organelles functioning at the immune synapse as well as presynaptic biogenesis in neurons (van der Beek et al., 2019; Edvardson et al., 2015; Sanderson et al., 2021; van der Welle et al., 2021). Failed recruitment and assembly of HOPS complex subunits on lysosomal membranes due to the defective interactions with small GTPase ADP‐ribosylation factor‐like protein 8B (Arl8b) is associated with impaired fusion of phagosomes with lysosomes and disturbed phagolysosomal maturation (Abo et al., 1982; Barry et al., 2012; Cox & Winchester, 2022; Garg et al., 2011; Khatter et al., 2015; Sasaki et al., 2013). It moreover delays presentation of a synthetic glycolipid, α‐galactosylceramide, to CD1d molecules in the lysosome and impedes microbial killing (Luzio et al., 2014). Immune deficiency caused by impaired development of NK cells and cytotoxic T cells has been documented in lysosomal biogenesis defects and abnormal endosomal‐lysosomal trafficking (Ballabio & Bonifacino, 2020; Ham & Billadeau, 2014; Orange, 2008). Finally, the severe nephritis with glomerular sclerosis and proteinuria is likely to be related to vacuolation and effacement of podocytes in which active endocytosis is impaired (Bechtel et al., 2013; Chen et al., 2013; Sofronova et al., 2022).

Unlike patients with the infantile form of mucopolysaccharidosis plus disease due to homozygosity for the p.Arg498Trp mutation in VPS33A, even by early adulthood, the patient P6, who is homozygous for the p.Arg200Pro mutation in the same region of the protein, has not hitherto developed full‐blown systemic complications such as glomerulonephropathy, broncho‐pulmonary complications associated with respiratory distress, or susceptibility to infections or spontaneous hemorrhage. Environmental factors, exposure to infections, vulnerability of the immune system in early childhood, or genetic background may explain the variable clinical penetrance in this monogenic disease and hence a mild clinical phenotype without the severe sporadic infections in genetically predisposed children (Abel et al., 2010).

At the biochemical level, however, urinary excretion of heparan and dermatan sulphate was raised in our patient, P6, after 20 years of age—as in patients with neonatal onset disease. Of special note, there was also an increased excretion of the non‐sulphated GAG, hyaluronic acid, in this patient homozygous for the p.Arg200Pro mutation (Figure 1f), and this abnormality may occur in infants with the p.Arg498Trp mutation. Hyaluronan receptors (stabilin‐1 and stabilin‐2) have been identified in electron‐dense vesicles in liver sinusoidal endothelial cells and colocalise with EEA1‐positive endosomes, suggesting that these receptors mediate clearance of endocytosed hyaluronic acid and possibly other GAG species—dermatan and heparan sulphates (Hansen et al., 2005). This observation suggests that accumulation of GAG is unrelated to the primary digestive function of lysosomal hydrolases but could result from abnormal intracellular trafficking via the endocytic pathway.

From the practical aspect, it is of paramount importance in the diagnosis of suspected lysosomal diseases with features of mucopolysaccharidosis, that advanced and sensitive methods for GAG analysis are used and that genetic studies should include *VPS33A* in relevant diagnostic panels. The clinical signs of intellectual disability with autism spectrum syndrome merit further studies to understand a role of the HOPS/CORVET complexes in impairment of cognitive skills development and mental retardation. The development of these disabilities in our patient immediately illustrates the need for diagnostic awareness combined with planning for care and clinical monitoring support over childhood and well into adult life. The occurrence of an attenuated VPS33A defect reported here for the first time in a young adult with late onset mucopolysaccharidosis plus disease and slowly progressive course limited to the skeletal system with psychomotor delay, clearly indicates a better prognosis for survival.

Fibroblasts obtained from patients with both forms of mutated VPS33A (p.Arg498Trp and p.Arg200Pro) show vacuolation and enlargement of endosomal and lysosomal compartments along the whole endocytic pathway. The diseased fibroblasts, moreover, showed enhanced fluorescence of Lysotracker dextrans, implying increased acidification and increased fluorescence of EEA1. Increased lysosomal acidification was attributed either to direct regulation of acidification (Kondo et al., 2017) or as a consequence of an altered balance of the fission and fusion events that can regulate the size and acidity of endosomal/lysosomal compartments (Bright et al., 1997; Pavlova et al., 2019). Just as had previously been noted in fibroblasts from VPS33A^p.Arg498Trp^ patients (Pavlova et al., 2019) we identified delayed trafficking of endocytosed lactosylceramide and its accumulation in endocytic organelles in the VPS33A^p.Arg200Pro^ patient. While this confirms impaired membrane trafficking, a feature observed in several sphingolipid diseases regardless of their primary cause, it relates to the effects on endosomal/lysosomal compartments and reflects the generally impaired function of this compartment in pathological conditions of lysosomal storage (Bright et al., 1997; Marks et al., 2013; Pagano, 2003; Pryor et al., 2006; Puri et al., 1999; Vruchte et al., 2004). Treatment with bortezomib significantly ameliorates delayed trafficking of fluorescent glycosphingolipid to the Golgi complex (Figure 5), providing evidence that an increased steady state concentration of the mutated VPS33A and hence, the assembled HOPS complex, improved intracellular endocytic membrane trafficking.

A recent study using a combination of light and electron microscopy techniques on ultrathin cryosections and systematic analysis of fluorescent organelles to compare with endolysosomal morphology, demonstrated that EEA1, usually regarded as an early endosomal marker was also present on a population of later endosomes after loss of Rab5, but where PI(3)P was still present (van der Beek et al., 2022). Thus, swollen EEA1‐positive organelles and increased LysoTracker fluorescence in the p.Arg200Pro and p.Arg498Trp mutant cells are likely to be related to the endosomes and lysosomes where the principal function of HOPS complex has been deficient. However, as previously shown in p.Arg498Trp fibroblasts (Kondo et al., 2017; Pavlova et al., 2019), there would be sufficient residual HOPS complex to bring about fusion of catalytically active lysosomes or autophagosomes in which uptake and delivery of fluorescent dextran to endosomes and lysosomes with a complement of active cathepsin B was unimpaired (Pavlova et al., 2019).

Our findings of accumulation of hyaluronic acid, dermatan, and heparan sulphates and increased EEA1 positive endosomes in patients with deficiency of HOPS and potentially CORVET complexes are intriguing and prompt further studies on the dynamic molecular interactions of particular GAG during endocytosis. Here we show that the two mutations described in VPS33A reduce the abundance of the protein and are also associated with diminished amounts of VPS16, another subunit common to the HOPS and CORVET complexes. This finding indicates that the complement of fully assembled HOPS complexes is decreased and that the interaction between VPS33A and VPS16 is necessary for the fusion of endosome and lysosome and autophagosome in living cells—as was found in knockout experiments previously conducted in vitro (Sofou et al., 2021).

The young man reported here has a relatively mild phenotype that might be accessible to empirical and ethically approvable therapeutic exploration with known repurposed drugs. It is noteworthy that substrate reduction therapy with eliglustat and miglustat (inhibitors of UGCG, which catalyze the biosynthesis of β‐d‐glucosylceramide, the precursor of gangliosides and cognate glycosphingolipids), improved glycosphingolipid trafficking in VPS33A^p.Arg200Pro^ fibroblasts (Figure 5). These findings immediately suggest that partial correction of the delayed turnover and accumulation of the glycosphingolipids in the lysosomal compartment can be achieved by inhibiting the *de novo* synthesis of gluco‐series sphingolipids (Cox et al., 2017; Pavlova et al., 2015). Increased glucosylceramide concentration may activate an alternative pathway that converts glucosylceramide to glycosylated cholesterol through transglycosylation catalyzed by intracellular GBA2 located outside of the lysosomal membrane (Boer et al., 2021; Ghisaidoobe et al., 2014; Marques et al., 2016). However, it remains unclear as to whether glycosylated cholesterol is formed in cells with VPS33A deficiency as a consequence of defective membrane sphingolipid trafficking.

In summary, we report a juvenile form of mucopolysaccharidosis plus disease caused by a hitherto unknown mutation p.Arg200Pro that affects a conserved residue in the VPS33A protein with an attenuated phenotype and neuropsychological complications manifested by late onset and slowly progressive natural course of this disease. Our study shows that the disease caused by deficiency of VPS33A and destablization of HOPS [and potentially CORVET complexes] is characterized by an expanded endocytic compartment and an increased proportion of acidic, swollen endolysosomes. Given the accumulation of GAG and glycosphingolipids due to defective endocytic traffic, we further showed that the approved proteasomal inhibitor, bortezomib, and inhibition of glycosphingolipid synthesis with the approved drugs, eliglustat or miglustat are plausible therapeutic options.

## CONFLICTS OF INTEREST

E. V. P: travel expenses—Global Challenge Research Fund, Research England (Inst); attendance and travel expenses to 2019 ESGLD Workshop—ESGLD. H. J. C.: virtual attendance to WORLD Symposium 2022—sponsored by Sanofi. T. M. C.: honoraria for lectures and program planning and support for unrelated investigator sponsored research from Takeda and Sanofi Aventis. The remaining authors declare no conflict of interest.

## Supporting information

Supporting information.
